# EP28 A case of ANCA vasculitis presenting with Sjögren’s serology

**DOI:** 10.1093/rap/rkaa052.027

**Published:** 2020-11-03

**Authors:** Shazeen Ayub, Marian Regan

**Affiliations:** Royal Derby Hospital, Derby, United Kingdom

## Abstract

**Case report - Introduction:**

Sjögren’s is an autoimmune multisystem disorder characterised by xerostomia, keratoconjunctivitis sicca and extra glandular manifestations. The presence of sicca symptoms helps with diagnosis but up to 20% patients do not have these. The prevalence of lung involvement has been reported up to 9-24% and includes changes such as NSIP and organising pneumonia. We present an interesting patient who had no sicca symptoms but positive immunology to suggest Sjögren’s. Changes in sequential CT chest scans were in keeping with Connective Tissue Disease- Associated Interstitial Lung disease (CTAILD). However, presentation with an acute renal injury resulted in a diagnosis of ANCA positive vasculitis.

**Case report - Case description:**

A 64-year-old Indian gentleman with medical background of controlled asthma was referred after 9 months of investigations for gluteal weakness. Initial blood tests included normal CK, ESR, CRP, vitamin B12, HbA1c and TFTs. Rheumatoid factor was low positive (20IU/ml), CCP negative. ANA was 1:80 with positive Ro, negative dsDNA. MRI of thighs was normal -no evidence of myositis. Nerve conduction studies showed no active denervation to suggest inflammatory myopathy and muscle biopsy showed myopathic features only. A CT scan revealed 3 small lung nodules recommending repeat scan.

When seen in rheumatology clinic, there was additional bilateral shoulder arthralgia with no reports of sicca symptoms or rashes. A repeat autoimmune screen and CT chest was sent. His ANA was 1:1280 with Ro antibodies; CT showed new ground glass changes (with old nodules). Organising Pneumonia was suggested, and respiratory opinion sought. Extended myositis screen was negative. When seen in respiratory clinic he had new haematuria- ANCA screen showed positive anti-PR3 antibody (23U/ml) with normal U+Es, urine PCR and CRP 8mg/L. Based on these results a renal biopsy was performed-which showed no obvious morphological abnormalities. Thus, a suspected diagnosis of CTAILD with Sjögren’s was made.

In clinic 2 months later, there was new shortness of breath and haemoptysis. Urine dip showed haematoproteinuria and Chest X-ray showed increase in peri-hilar masses. He was admitted urgently- blood tests showed a decline in renal function- urea 26.8mmol/L, Creatinine 838umol/L, CRP of 435mg/L and Haemoglobin 100g/L. Urgent repeat renal biopsy was done and CT thorax showed deterioration with bilateral consolidation and new lung lesions. Urgent Plasma exchange and dialysis (9 cycles) was given. Initial results from renal biopsy showed presence of crescents and he was started on cyclophosphamide. On this kidney function has improved- urea 18.4mmol/L and Creatinine 302umol/L; he remains on oral cyclophosphamide.

**Case report - Discussion:**

With ongoing symptoms, an underlying autoimmune cause of his symptoms was felt likely. However inflammatory markers remained normal and so did CK. Despite this MRI and muscle biopsies were performed - which again were normal. The only tests that were positive were immunology (Ro antibodies) and a CT chest (showing initial small lung nodules). These findings pointed toward CTAILD with Sjögren’s being the likely diagnosis.

As new lung nodules were seen, repeat CT scans were done- which showed gradual interstitial changes- the main radiological differential diagnosis was Organising pneumonia. Further investigations were delayed due to patient travel and COVID, but ongoing respiratory advice was sought. Even with changes in CT findings the patient remained stable with normal inflammatory markers. However, the clinical picture changed quite rapidly over a month (despite 2+ years of previous symptoms) with presentation of pulmonary-renal vasculitis. Plasma exchange and dialysis were given. A good response with a positive renal biopsy confirmed the most likely diagnosis was Granulomatosis with Polyangiitis (GPA).

This case was interesting- the main complaint was of myopathy with no physical signs. Despite this biopsy were performed (muscle and kidney), which were all normal. The red herring in his case was a normal renal biopsy- steering us in the direction of CTD-AILD instead of GPA.

**Case report - Key learning points:**

In patients with clear symptoms matching their investigations the diagnosis is often obvious. When this is not the case and symptomatology does not match results (i.e. no Sicca symptoms but positive immunology to suggest Sjögren’s) suspicion should remain high. Multidisciplinary working can provide insight, which this case does highlight-with input required from Neurology, Respiratory and Renal medicine.

Negative results should be taken in context with patients and their symptoms. Initial renal biopsy in this case was normal- however after a review, further comments were made on the sparsity of glomeruli in the sample. Therefore, tissue obtained for diagnosis should always be questioned and clinical suspicion should remain high. In addition, repeat investigations (6 monthly CT scans) can help note any interval change.

Thorough history and examination in follow up of patients can help look out for evolving changes. With the new symptoms of haemoptysis and haematoproteinuria this pointed us to the eventual diagnosis. The road to diagnosis in this case was prolonged with an acute drop in kidney function and pulmonary haemorrhage needing urgent Plasma exchange and dialysis. Thankfully, the patient continues to make a good recovery.

A last point to add is although isolated myalgia has been described as a presentation of systemic vasculitis in the literature, those patients have had elevated CK and positive muscle biopsy. Our patient did not have any positive findings with over 2 years of symptoms. Therefore, we feel this case was unique in presentation and has valid learning points as above.

